# The roles of amygdala subnuclei in processing of approaching in- and outgroup others in virtual space

**DOI:** 10.1093/scan/nsaf119

**Published:** 2025-11-13

**Authors:** Gloria Mendoza-Franco, Olli Tammilehto, Inga Jasinskaja-Lahti, Matthias B Aulbach, Ville J Harjunen, Anna Peltola, Niklas Ravaja, Matilde Tassinari, Saana Vainio, Iiro P Jääskeläinen

**Affiliations:** Department of Neuroscience and Biomedical Engineering, Brain and Mind Laboratory, Aalto University School of Science, Espoo, 02150, Finland; Department of Neuroscience and Biomedical Engineering, Brain and Mind Laboratory, Aalto University School of Science, Espoo, 02150, Finland; Faculty of Social Sciences/Social Psychology, University of Helsinki, Helsinki, 00100, Finland; Department of Psychology, Centre for Cognitive Neuroscience, Paris-Lodron-University of Salzburg, Salzburg, 5020, Austria; Department of Psychology and Logopedics, University of Helsinki, Helsinki, 00100, Finland; Department of Neuroscience and Biomedical Engineering, Brain and Mind Laboratory, Aalto University School of Science, Espoo, 02150, Finland; Department of Psychology and Logopedics, University of Helsinki, Helsinki, 00100, Finland; Faculty of Social Sciences/Social Psychology, University of Helsinki, Helsinki, 00100, Finland; Department of Neuroscience and Biomedical Engineering, Brain and Mind Laboratory, Aalto University School of Science, Espoo, 02150, Finland; Department of Neuroscience and Biomedical Engineering, Brain and Mind Laboratory, Aalto University School of Science, Espoo, 02150, Finland

**Keywords:** amygdala, intergroup threat, amygdala subnuclei, interpersonal distance

## Abstract

Activity in the amygdala during intergroup contact and prejudice has been explained as a conditioned threat response and as a social saliency response. More recently, the theory that both explanations are true has received some empirical support, suggesting that the amygdala subnuclei (basal, lateral, and central) may play distinct roles in the social categorization process. In this study, we used machine learning to decode amygdala subregion activations during simulated encounters with protagonists representing four different stereotypes. In these encounters, a generalized threat was presented: the protagonists initially remained at a distance, then approached the participant, and finally entered their personal space. Using a time-resolved decoding approach, we studied the effect of interpersonal distance on the subnuclei’s prejudice response. Moreover, a second classification was used to evaluate how the group relevance modified the perceived generalized threat. Both classifications revealed that all amygdala subregions encoded stereotype content. The likelihood of successful stereotype classification in each subregion was modulated differently by the context. Methodologically, our results show that a time-resolved decoding approach provides tools for studying prejudice within the amygdala’s subnuclei. Neuroscientifically, our results support the theorization of different and parallel functions of amygdala subnuclei in prejudice.

## Introduction

The social neuroscience of prejudice explores the mechanisms underlying social evaluations, which determine how we perceive and engage with members of specific social groups. Specifically, prejudice refers to the evaluation or emotional response to individuals from a particular group based on preconceived beliefs (e.g. stereotypes) (Amodio 2014), and it is part of the social categorization process that distinguishes “us” (ingroup) from “them” (outgroup). While this cognitive function is essential for our survival as social beings, it is also the origin of intergroup conflict (Durrheim et al. 2016, Amodio and Cikara 2021). Social neuroscience of prejudice offers the opportunity to connect neuroscientific research with some of the most pressing social issues and challenges we face as a society. A deeper understanding of the mechanisms of prejudice and stereotyping could help in the development of effective interventions to reduce the escalation of intergroup conflicts (Cikara and Van Bavel 2014).

### Prejudice and intergroup threat

According to the intergroup threat theory (ITT), attitudes towards outgroup members, particularly negative ones such as prejudice, are explained by the perception of different intergroup threats (Stephan and Stephan 1996, Stephan et al. 2015). ITT categorizes intergroup threats into realistic threats (e.g. competition for resources) and symbolic threats (e.g. threats to values and beliefs) (Aberson and Gaffney 2009, Stephan et al. 2015). Multiple studies have provided evidence supporting ITT, demonstrating a correlation between negative attitudes towards outgroups and the perception of either realistic or symbolic threats across a range of social groups (for reviews, see Riek et al. 2006, Rios et al. 2018). Notably, the perception of threat is sufficient to trigger or amplify intergroup bias, regardless of the actual existence of such a threat (Stephan et al. 2015, Chang et al. 2016). Furthermore, it is suggested that different kinds of threats elicit distinct emotional responses; for instance, some groups may be perceived as fearful, while others may elicit anger, pity, or disgust (Cottrell and Neuberg 2005).

Another theoretical model that proposes diverse emotional reactions elicited by prejudice is the stereotype content model (SCM) (Cuddy et al. 2007, Fiske et al. 2007). The SCM states that stereotypes share common content across two dimensions: warmth and competence. Consequently, intergroup encounters prompt two fundamental appraisals: first, whether outgroup members have positive or negative intentions (warmth, e.g. ‘Do they intend to help or harm me?'); and second, whether they possess the ability to act on those intentions (competence, e.g. ‘Are they capable of doing so?') (Cuddy et al. 2009). Thus, SCM provides a more nuanced categorization of social groups compared to the traditional ingroup/outgroup approach (Cuddy et al. 2009). According to SCM, the four main stereotypes are commonly associated with specific emotions: competent and warm groups elicit admiration, competent and cold groups evoke envy, incompetent and warm groups produce pity, and incompetent and cold groups elicit contempt (Cuddy et al. 2007). Altogether, the ITT and SCM provide a rich, complex framework to study intergroup dynamics and prejudice.

### The role of the amygdala in prejudice

Previous research has identified multiple brain regions associated with prejudice (for reviews, see Amodio 2008, Cikara and Van Bavel 2014, Amodio and Cikara 2021). Among them, the amygdala was early on considered a good candidate to study, as it has been traditionally viewed as a central part of emotional processing, particularly fear conditioning (Adolphs et al. 1994, Murphy et al. 2003, Tovote et al. 2015). Early research found differential amygdala activity while perceiving ingroup vs. outgroup faces (Hart et al. 2000, Phelps et al. 2000, Cunningham et al. 2004, Wheeler and Fiske 2005) and correlations between amygdala activity and implicit prejudice attitudes (Phelps et al. 2000, Cunningham et al. 2004). These insights, along with the theoretical association of prejudice with threat, led to the interpretation of amygdala involvement in intergroup categorization as indicative of a potential threat response (Wheeler and Fiske 2005, Chekroud et al. 2014, Lantos and Molenberghs 2021, Saarinen et al. 2021). Supporting this intergroup threat interpretation, amygdala activity has also been associated with learnt negative stereotypes of social groups (e.g. Black males) (Lieberman et al. 2005, Ronquillo et al. 2007, Senholzi et al. 2015), the direct gaze of outgroup members (Richeson et al. 2008), and the activation of negative racial stereotypes through priming (Forbes et al. 2012). All of these conditions are considered social threats (Maner et al. 2005, Trawalter et al. 2008).

However, the threat-based interpretation has been challenged by contradictory results regarding amygdala activation in intergroup scenarios. For instance, multiple studies have failed to find differential amygdala activation during intergroup encounters (Phelps et al. 2003, Knutson et al. 2007, Schreiber and Iacoboni 2012, Brosch et al. 2013). Furthermore, amygdala activation disappears when participants engage in a secondary task or their social goals shift (Lieberman et al. 2005, Wheeler and Fiske 2005, Van Bavel et al. 2008). Importantly, the amygdala responds not only to negative stimuli but also to positive stimuli and any socially or personally relevant information (Hamann et al. 2002, Zald 2003, Cunningham et al. 2008, Cunningham and Brosch 2012, Varkevisser et al. 2024). As part of the saliency network, the amygdala contributes to the processing and labelling of relevant stimuli (Sander et al. 2003), including socially relevant information (Varkevisser et al. 2024). Social saliency is continuously and rapidly changing, as it is modulated by context, social goals, and culture (Cunningham and Brosch 2012, Chang et al. 2016). According to the social saliency interpretation, the amygdala’s involvement in prejudice is related to the appraisal of social relevance, with outgroup membership, race, or intergroup threat being sometimes the most salient information during intergroup interactions, explaining its role in social categorization (Cikara and Van Bavel 2014).

While the hypothesis that social saliency may drive the prejudice-related amygdala activation is plausible, the involvement of fear conditioning in prejudice cannot be ruled out (Amodio 2014, Amodio and Cikara 2021). It has been suggested in the theory that different amygdala subnuclei are activated by distinct processes associated with prejudice, which could explain the inconsistencies in experimental results (Amodio 2014, Chekroud et al. 2014). To simplify the functioning of the amygdala subnuclei, the lateral subnucleus (LA) receives information from the sensory organs. If the stimulus is considered threatening, the central nucleus (CeA) will be activated; otherwise, the basal nucleus (BA), which has an instrumental function, is activated (LeDoux 2012). Multivariate pattern analysis (MVPA) techniques have been used to investigate the functioning of amygdala subnuclei (Bach et al. 2011, Hrybouski et al. 2016), as the method has been proven effective in the detection of differential cognitive and affective states, despite the small size, when traditional univariate analysis did not yield significant results (Bach et al. 2011, Vizioli et al. 2018). However, research focused on the role of amygdala subnuclei in prejudice is limited. For instance, Izuma et al. (2019) found that medial (CeA) and lateral (BA and LA) regions of the amygdala could have distinct functions related to implicit prejudice, as they differentially allowed for the classification of implicit evaluations towards ingroup/outgroup members. To our knowledge, the use of MVPA to study amygdala subnuclei participation in prejudice response when confronting more than two groups is lacking.

In this research, we used functional magnetic resonance imaging (fMRI) data from amygdala subnuclei (specifically, LA, BA, and CeA, and the whole amygdala) to decode SCM stereotypes during a simulated encounter. We showed participants 360-degree videos featuring protagonists from stereotyped groups who approached them and invaded their personal space. Previous research indicates that physical proximity to outgroups enhances negative attitudes and prejudice (Xiao et al. 2016). Therefore, we expected the decoding performance to be modulated by interpersonal distance.

We hypothesize that each subnucleus classifier will perform differently at different points in the stimulation. We expect the LA classifier to perform best at moments that are more relevant from a perceptual point of view, e.g. the first seconds of the encounter or when there are behavioural changes. For the BA classifier, we expect better performance during the first half of the encounter, when the protagonist is at a distance and the stimulation is not necessarily considered threatening but socially relevant. Finally, we anticipate the opposite situation with the CeA classifier; better performance during the approaching movement would indicate the involvement of a conditioned fear response elicited by the reduction of interpersonal distance.

To further explore the differential effect of interpersonal distance in the amygdala subnuclei between stereotypes, we performed a second classification. The second classifier aimed to predict the interpersonal distance using data from each stereotype. We expected a modulation of this second classifier’s performance driven by the relevance of each group.

## Materials and methods

### Selection of SCM groups in an initial online study

**Figure 1.**
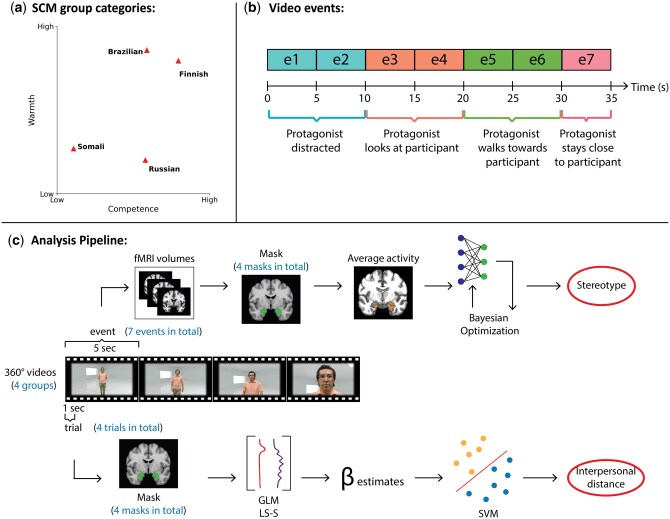
Details on the experimental design and analysis stages. (a) Group stereotypes used as stimulation for the fMRI experiment. (b) Description of the video events. We used a time window of 5 s to study only data within each event. (c) Stages of the analysis. Two classification tasks were performed.

To define which groups to use in the fMRI experiment, we implemented an online survey using the SCM questionnaire from Cuddy et al. (2009), translated into Finnish. The survey was completed by 111 participants, ages 17–78 years (68% female, 28% male). All the details about the design, application, data analysis, and power analysis of the survey are documented in the online supplementary material, Section A. From the results, four nationalities were selected as they represented different levels of perceived competence and warmth (see Fig. 1a).

Finns, the ingroup, correspond to the high-competent/high-warm group and were perceived as more competent than Brazilians, Russians, and Somalis (*P* < .001) and warmer than Russians and Somalis (*P* > .001). Brazilians are in the medium-competent/high-warm group, as they were considered more competent than Somalis (*P* > .001) and warmer than Russians and Somalis (*P* < .001). Russians represent the medium-competent/low-warm group and Somalis the low-competent/low-warm group. The survey results did not show any group perceived as low-competent/high-warm or high-competent/low-warm, which is why we selected groups with medium competence (Brazilians and Russians). To confirm that the fMRI participants perceived the four groups similarly, the same SCM questionnaire was administered after scanning.

### fMRI study participants

FMRI measurements were collected from 42 individuals (17 males, 23 females; ages 21–54 years; mean age 30.4 years) participating in a task simulating an intergroup encounter (the virtual encounter task). All the participants were native Finns with Finnish parents, and the cohort was independent from the online survey. All participants were healthy, had normal or corrected-to-normal vision, and provided informed consent prior to participation. Aalto University Research Ethics Committee reviewed and approved the experimental protocol.

### The fMRI virtual encounter task

To simulate an intergroup encounter, 35-s-long 360-degree videos were shown to participants inside the scanner. The videos featured the following uniform narrative (see Fig. 1b): first, the protagonist is shown in a room being distracted (Events 1–2). Then they lock eyes with the participant (Event 3–4). Next, the protagonist approaches the participant until they are intimately close (their face occupies 80% of the display) (Events 5–6). Finally, the protagonist stares at the participant while being uncomfortably close to them (Event 7). Each narrative event lasted for 5 s. Before each video, a short description of the protagonist, showing their name and nationality, was displayed for 4 s. After each video, a 10–12 s washout period was initiated before the next stimulus video. The stimulus set consisted of 40 videos, with two male actors per nationality/stereotype content category. Five clips were generated for each actor, using different footage and neutral backdrops. Additionally, participants were told they all lived in the Helsinki region. The simulated encounter task was designed to introduce two types of threats: an intergroup threat by depicting members of differently stereotyped categories and a generalized threat by manipulating the interpersonal distance.

### fMRI data acquisition

All fMRI data were acquired at the Advanced Magnetic Imaging Centre at Aalto University, using a Siemens 3.0 T Magnetom Skyra whole-body scanner with a 30-channel head-neck coil. Both anatomical and functional scans were collected for each participant. A T1-weighted MP-RAGE sequence was used to acquire high-resolution anatomical images, with a voxel size of 1.0 × 1.0 × 1.0 mm. Functional images of the whole brain were collected utilizing a T2*-weighted gradient-echo echo-planar imaging sequence with the following parameters: 54 slices, TR = 1.6 s, TE = 30 ms, flip angle = 70°, matrix size = 256 × 256, voxel dimensions = 3.0 × 3.0 × 3.0 mm, and field of view (FOV) = 512 × 512 mm^2^.

### MRI data preprocessing

Anatomical and functional data were preprocessed using fMRIPrep 23.2.0a2 (Esteban et al. 2019). T1-weighted images were corrected for intensity non-uniformity, followed by skull-stripping. From the stripped images, brain tissues were segmented into grey matter, white matter (WM), and cerebrospinal fluid (CSF) using FSL. Individual participants’ images were spatially normalized to standard MNI152 space [MNI152NLin2009cAsym from Fonov et al. (2009)] via nonlinear registration.

Functional images were head-motion and slice-time corrected, then co-registered to the T1 images. Finally, the functional BOLD data from individual subjects were resampled onto the standard MNI152 space. Confounds related to head motion, WM, CSF, and the whole brain were calculated from the preprocessed BOLD using high-pass filtering and principal components analysis.

### Regions of interest

Four regions of interest (ROIs) were selected for the classifications: LA, BA, CeA, and the combination of the previous three. The ROIs were masked using the subregion segmentation algorithms by Saygin et al. (2017) and Nilearn 0.10.2 (Abraham et al. 2014) tools. Voxel signals were corrected for confounds, masked, and normalized to a zero mean and unit variance. The data were not smoothed due to the small size of the ROIs: LA = 65, BA = 41, CeA = 5, and the combination mask = 111.

### Group classification

The group classification was repeated for each ROI and each event (see Fig. 1c), leading to a total of 28 classifiers (4 ROIs × 7 events). All classifiers were optimized using Bayesian optimization. Analyses were conducted directly at the group level with leave-one-participant-out cross-validation (Wang et al. 2020). A total of 1680 (42 participants × 10 videos × 4 categories) samples were used for each classification. Previous research has suggested using at least 200–400 samples per category for fMRI data classification (Cui and Gong 2018, Jollans et al. 2019); our study uses 420 samples per category. Scikit-learn (Pedregosa et al. 2011) and TensorFlow’s Keras software libraries were used to train, validate, and test the classifiers.

The purpose of this first classification was to track the classification accuracy over time for each ROI so that any changes could be compared with variations in interpersonal distance. To reduce computational costs, fMRI volumes within 5-s time windows (denoted as events in the text) were averaged and used as features rather than individual volumes. Because the narrative in the videos developed slowly, we believe that a time window is sufficient to detect changes in the separability of patterns at the key moments. Using scan-by-scan classification without a first-level general linear model (GLM) regression was considered appropriate for this analysis to preserve the time component of the signal. Moreover, fitting a GLM regression while considering each volume as a separate regressor in continuous data has been found to create high-variance estimates, harming the classification (Loula et al. 2018). The classification of scan-by-scan continuous data is referred to as time-resolved decoding (Ariani et al. 2018) and has been used in similar studies that aimed to track classification accuracies over time (Soon et al. 2008, Harrison and Tong 2009, Gallivan et al. 2013, Chan et al. 2020). A delay of 6 s was added to the stimulus onset to account for the haemodynamic response delay (Chan et al. 2020).

All classifiers consisted of fully connected multilayer perceptrons (MLP) without hidden layers, a linear activation function, L2 regularization, and an Adaptive Moment Estimation (Adam) optimizer (Kingma and Ba 2014). MLPs without hidden layers that use linear activation functions are essentially linear classifiers and have previously been able to classify emotional responses from the brain during video viewing (Saarimäki et al. 2016, Mendoza-Franco et al. 2025). Only linear classifiers were used in this study, as their performance does not seem to differ from non-linear classifiers on functional fMRI data for sample sizes below 10 000 participants (Schulz et al. 2020).

All classifications were repeated 1000 times with initial random weights, and final ROC area-under-curve accuracies were *t*-tested against the chance level (0.25). A 1000-label permutation test was used to verify the dependency between features and labels.

### Interpersonal distance classification

The interpersonal distance classifier was trained with data extracted from the first second of events that represent a change in interpersonal distance (Events 1, 3, 5, and 7) (see Fig. 1b and c). This classification uses data from each group and ROI across events. A total of 16 classifiers (4 groups × 4 ROIs) were evaluated using 1680 samples (4 events × 10 videos × 42 participants) and 420 samples per category.

A first-level GLM regression was performed with a Least Squares–Separate (LS-S) approach. The LS-S method considers two regressors for each sample: one corresponding to the trial of interest and a second one that combines all the other trials as a nuisance regressor. This approach has shown good performance in estimating samples for MVPA analysis with short intertrial intervals (Mumford et al. 2012); the time intervals between distance samples are 9 s long. The beta estimates were used as features for the classification.

Support vector machines with a linear kernel were used for the classification. Accuracy levels were obtained using a 10-fold cross-validation with random initial weights between participants. The classifications were repeated 1000 times, and mean accuracies were *t*-tested against the chance level (0.25). A 10 000-label permutation test was used to verify the dependency between features and labels. A Dunn test was used to contrast the classifiers’ performance between groups across ROIs.

## Results

### Behavioural results

Results from the post-test questionnaire confirmed that the fMRI participants shared the same group stereotypes as those of respondents in the online questionnaire. The detailed results and statistical test are shown in the online supplementary material, Section B.

### Group classification

**Figure 2.**
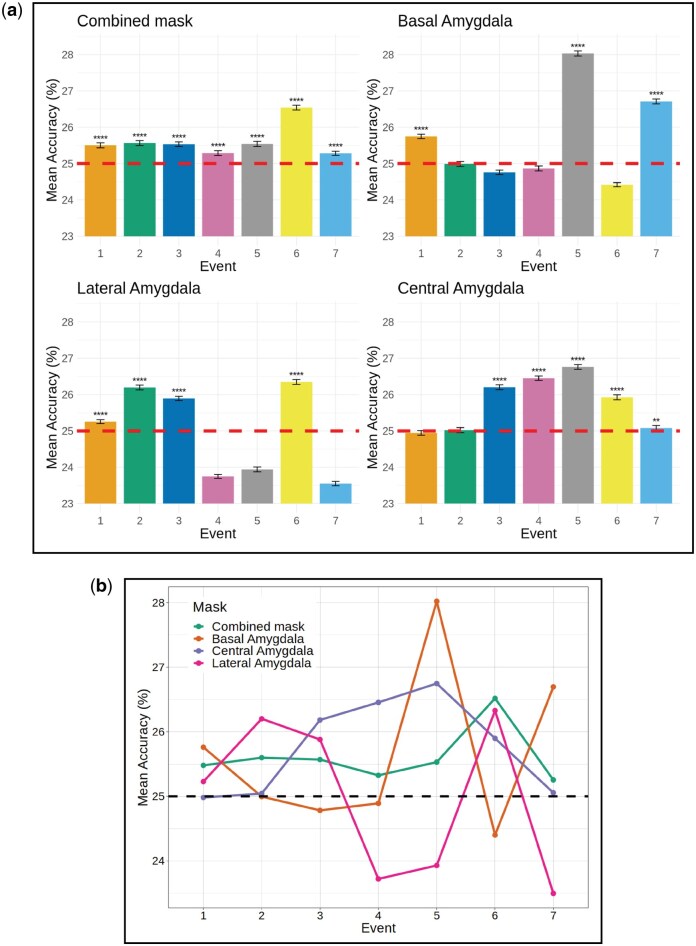
Estimated mean classification accuracies for the group classification. (a) Mean accuracies for each event and their confidence intervals for the best-hyperparameterized models for each subregion-event classifier pair in the outgroup classification task (**** = P < .0001, ** = P < .01, Bonferroni corrected). (b) All the mean accuracies in the same plot to allow comparison. The dashed horizontal line represents the chance-level (1/4 = 0.25) in the group membership classification task.

Cross-validated performance estimates showed statistical significance for all the classifiers with mean accuracies above chance level (*P* < .0001, Bonferroni-corrected; see Fig. 2). The combined mask performed above chance level across all events, while the other ROIs failed the classification in some events. Mean accuracies ranged from 0.235 to 0.280.

**Figure 3.**
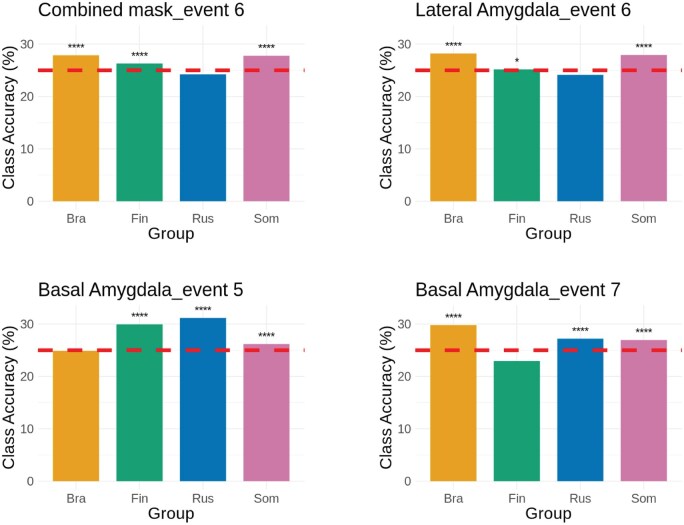
Class accuracies for the classifiers with significant performance and their significance. The dashed line represents the chance level (25%), (**** = P < .0001, * = P < .05, Bonferroni corrected).

Class accuracies for classifiers across events and ROIs are shown in Fig. 2a. Estimated ROC area-under-curve scores for significant classifiers ranged from 0.502 to 0.529, meaning that predictions made by the classifiers were above chance level, yet not robust in terms of effect sizes. Results from the label permutation test were significant for Event 5 in BA (accuracy = 0.282, *P* < .01), for Event 6 in the combined region (accuracy = 0.272, *P* < .05) and LA (accuracy = 0.271, *P* < .05), and for Event 7 in BA (accuracy = 0.282, *P* < .05). Class accuracies of those classifiers with significant results in the label permutation test are reported in Fig. 3. Russians, categorized as the medium-competent/low-warm group, were the only class that was not predicted above chance by the combined mask and the LA. On the other hand, for the BA, the groups perceived as warm (Brazilians and Finns) were the classes predicted below the chance level.

### Classification of interpersonal distance

All classifiers achieved statistically significant mean accuracies (*P* < .0001, Bonferroni-corrected) above the chance level (0.25), according to cross-validation results. Mean accuracies ranged from 0.264 to 0.335. ROC area-under-curve scores ranged from 0.51 to 0.59. The label-permutation test was significant for all classifiers (*P* < .05), except for the BA classifier using the medium-competent/high-warm group’s (Brazilians) data. Class accuracies for all classifiers are shown in the online supplementary material, Section C.

**Figure 4.**
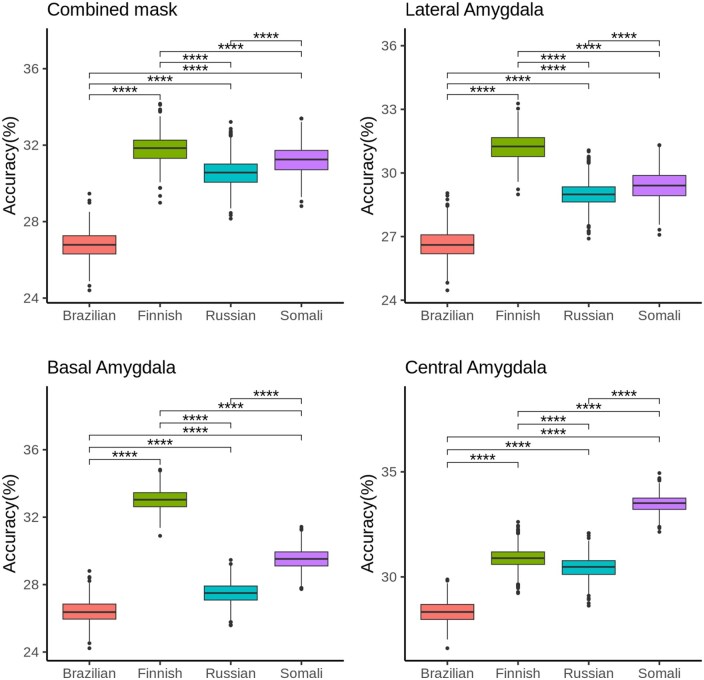
Comparison of classifiers within each ROI. Each boxplot corresponds to the corresponding classifier’s mean accuracies from the 1000 cross-validation runs. **** means significant (*P* < .0001, Bonferroni corrected).

When comparing the classifiers within each ROI, significant differences were observed (see Fig. 4). Classifiers trained on ingroup data performed best in the LA, BA, and combined regions. Meanwhile, the low-competent/low-warmth group classifier had the best performance within the CeA region. The medium-competent/high-warm classifiers performed worst across all ROIs.

## Discussion

Previous research has suggested that the amygdala plays a role in the social categorization process and prejudice (Amodio 2014, ­Amodio and Cikara 2021). Notably, there has been debate over the interpretation of amygdala activity during intergroup contact. The threat-based interpretation associates prejudice with a Pavlovian conditioned fear response towards threatening outgroups (Wheeler and Fiske 2005, Chekroud et al. 2014). On the other hand, the social saliency interpretation argues that the social relevance of groups drives amygdala activity (Cikara and Van Bavel 2014). Adding to this debate, some authors have suggested that both theories can be true and that different amygdala subnuclei are activated by different processes associated with prejudice (Amodio 2014, Amodio and Cikara 2021). The possibility of this parallel function of amygdala subnuclei has recently received empirical support (Izuma et al. 2019). The purpose of this study was to investigate the decoding of stereotype content within the LA, BA, and CeA subnuclei and the whole amygdala. Moreover, the modulation of the stereotype pattern’s separability by generalized threat (e.g. reduced interpersonal distance) was tested. Finally, we compared the effect of interpersonal distance reduction on amygdala activity between stereotypes within each ROI.

Results from the group classification show that all ROIs are involved in the categorization of stereotypes. The combined region achieved the best performance, successfully classifying all groups in all video events. Meanwhile, the subnuclei classifiers were ­successful only for some events. Interestingly, the best and worst performances of each subnucleus classifier were observed during distinct video events. These results support the hypothesis that distinct subnuclei have different functions in prejudice, showing greater pattern separability at different moments of the videos. Notably, the successful performance of the combined mask across events shows that data from the whole amygdala can reflect a confounded version of the subnuclei activity patterns, which would explain the contradictory findings in previous studies.

We predicted that the LA classifiers would perform better on events with behavioural changes, as these would be more relevant from a sensory perspective. The results showed better performance during the first three events (e1, e2, and e3) and e6, which shows the protagonist coming close to the camera. The basolateral complex, and particularly the LA nucleus, is considered a site of sensory convergence (Amodio 2014). Its function has been implicated in the processing of sensory information, fear learning, fear memory, and fear extinction (LeDoux 2013). In particular, LA activity has been associated with the evaluation of new or ambiguous stimuli (Grosso et al. 2018), which explains why the initial moments of the videos were particularly relevant for LA pattern separability. Moreover, the protagonist’s approach to the camera, particularly when becoming very close (e6), is an ambiguous stimulus, as participants had no information about the protagonist’s intentions. Our hypothesis was partially supported; we expected good performance in e5 (when the approaching starts) rather than in e6. Notably, multiple factors can explain low classification accuracies. It could be that the stimulation was not strong or relevant enough to activate the ROI; alternatively, the LA response to the stimulation may have been similar across all the stereotypes, preventing classification. Watching someone approach us represents a potential threat that needs to be evaluated, probably by LA. Hence, it would be pertinent to suggest that the evaluation of the potential threat was prioritized over the evaluation of the stereotype content in e5.

The BA group classifiers were successful in events e1, e5, and e7. These results reject our hypothesis, which predicted better performance at the beginning, before the interpersonal distance was reduced. Research has associated BA with the processing of safety and reward learning, as well as with goal-directed behaviour (Parkinson et al. 2000, LeDoux 2013, Sangha et al. 2013). High classification accuracy means that BA activation patterns for each stereotype were separable or distinct. Hence, it is possible that different stereotypes elicited a distinct perception of safety in e1, e5, and e7. Indeed, the first impression (e1), the moment the protagonist begins to approach (e5), and the protagonist’s stare when standing very close (e7) are instances in which social categories become relevant to the evaluation of safety (or its absence). As per our design, we would expect a similar result in e3 (when the protagonist locks eyes with the participant); however, it is possible that, given the protagonist’s distance, the locking of eyes was less impactful than other changes in behaviour.

Research has found a clear association between CeA activity and fear conditioning (Davis 2006, LeDoux 2013, Hrybouski et al. 2016, Fadok et al. 2018). CeA classifiers’ performance across events confirms our predictions of high performance during the approach of protagonists. In fact, CeA classifiers can distinguish stereotypes from the moment protagonists lock eyes with the participant until the end of the video. From ITT and the relationship between distance and prejudice, we know that each stereotype represents different social threats, and the reduction of distance is considered a threat or not, depending on the stereotype. Consequently, the separability of stereotype patterns within CeA during the second half of the videos may reflect differential threat responses to the same threat across stereotypes. This finding demonstrates the complexity of intergroup threat, as the classifiers distinguished the threat responses evoked by four groups with multiple contextual cues.

When comparing the predictability of interpersonal distance between stereotypes, the ingroup showed the best performance in the whole amygdala, LA, and BA. This result suggests a greater impact of the interpersonal distance reduction on the ROIs’ activity when encountering ingroup members. Meanwhile, the low-competent/low-warm group, the most extreme outgroup, led to greater separability of distance patterns in CeA. It is plausible to associate the separability of distance patterns within ROIs with the relevance of the encountered group. It appears, then, that the ingroup is the most relevant or salient group for LA, BA, and the whole amygdala, and the low-competent/low-warm group is more relevant for CeA. This interpretation is consistent with the proposition that distinct amygdala subnuclei process social saliency and social threat. According to our results, social saliency may drive the LA and BA prejudice responses, while social threat may drive the CeA prejudice response. Interestingly, the medium-competent/high-warm group had the lowest accuracy across all ROIs. According to the SCM, the combination of perceived low competence and high warmth forms the ambivalent paternalistic stereotype (Cuddy et al. 2009). Within this stereotype, groups typically considered incapable of harm are categorized (i.e. senior citizens or disabled people). The perception of the medium-competent/high-warm group as less threatening than the others may explain the poor performance of the distance classifiers.

Our study made two significant contributions. First, it showed that all three tested ROIs, LA, BA, and CeA, encode stereotype content; hence, they play a role in prejudice. The separability of stereotype-activation patterns within the amygdala subnuclei depends on contextual cues and the potential presence of threat, suggesting different functions within the social categorization process. In particular, we suggest that social saliency drives prejudice in LA and BA, while social threat drives prejudice in CeA. Second, the experimental design featured four stereotyped groups and a generalized threat, providing richer, more realistic stimulation than typical designs. We believe that studying the dynamics of intergroup threat at the neural level over time is a promising path to follow for a deeper understanding of prejudice.

As an overall critical note, the classification accuracies barely exceeded the chance level. This could have resulted from several factors, including the small size of amygdala subregions, which can challenge the spatial resolution of fMRI; misalignments between MNI152 template-based masks and individual data; and/or an experimental task that was not threatening enough, especially since the flow of events repeated across trials. The choice of classifier type and parameters could have also affected the results. It is also important to note that the interpretability of accuracy values is limited, as they do not indicate how different or similar activation patterns are. More research is necessary, particularly using a standard mass-univariate approach, to quantitatively test and compare neural activations in similar conditions. Nevertheless, despite these limitations, the results from this study provide promising evidence of the use of time-resolved decoding to study the activity of amygdala subnuclei during intergroup encounters over time.

## Conclusion

In summary, stereotyped groups and interpersonal distance could be classified above chance level from amygdala subnuclei data. A modulation of the classification accuracy of stereotypes by the reduction of interpersonal distance suggests that the LA, BA, and CeA have distinct roles in the amygdala’s prejudice response. The relevance of the ingroup for the interpersonal distance classification in LA and BA, and of the low-competent/low-warm group in CeA, supports the theorization of two mechanisms of prejudice within the amygdala. The results suggest a social saliency response in LA and BA and a threat response in CeA during the social categorization process.

## Supplementary Material

nsaf119_Supplementary_Data

## Data Availability

Access to the data and code that corroborate the findings of this research is available upon a reasonable inquiry.
