# Supplementary material for: The roles of amygdala subnuclei in processing of approaching in- and outgroup others in virtual space
Source: Soc Cogn Affect Neurosci. 2025 Nov 13;20(1):nsaf119. doi: 10.1093/scan/nsaf119 (PMC12673210; doi:10.1093/scan/nsaf119)
Supplement: nsaf119_Supplementary_Data [file nsaf119_supplementary_data.pdf]

Supplementary Materials for

The roles of amygdala subnuclei in processing of approaching in- and outgroup others in virtual space

A. Online SCM Survey

To define which groups to use in the fMRI experiment, we implemented an online survey using the SCM questionnaire from Cuddy et al. (2009), translated into Finnish. Results were analysed only at the group level, projecting the median ratings of each group onto a two-dimensional map. It is common to form clusters of evaluated groups and compute differences between clusters using t-tests. In our case, as we planned to use the groups as stimuli rather than further analyse the stereotyping context in Finland, we performed non-parametrical tests only between the groups that appeared to be good candidates for use as stimuli and skipped the cluster formation. The criteria for selecting a group candidate were the availability of enough actors from all groups to produce the stimuli and the maximization of differences in warmth and competence perception. The ideal selection of groups would include one group within each stereotype, considering the Finnish ingroup as the HC-HW group.

Materials

In this study, a total of eleven nationalities were evaluated in terms of perceived competence and warmth. The nationalities included in the survey were selected considering the availability of actors' ethnicities and the relevance of each group in the Finnish context (e.g., Brazilians are the largest Latin American group in Finland). The online survey consisted of eleven pages, with each page displaying a photograph of a member of each group, along with their nationality. All photographs featured neutral facial expressions and a solid-colored background. Following each photograph, participants were required to complete the SCM questionnaire. It was necessary to complete the evaluation of the presented group before moving forward to the next one. The order of groups was randomized each time.

The questionnaire, as utilized in Cuddy et al. (2009), includes four questions that evaluate each dimension (e.g., competence and warmth). All items were rated on a five-point scale (1 = not at all, 5 = extremely). The wording of all questions was the same: *As viewed by Finnish society, how (adjective) are members of this group?* The adjectives *competent*, *confident*, *capable*, and *skillful* were used to evaluate the perception of competence, while *friendly*, *warm*, *good-natured*, and *sincere* were used to evaluate the perception of warmth.

Participants

According to previous studies (Cuddy et al., 2009), the mean effect size of significant differences in perceived competence or warmth between groups was 0.8, $p < 0.01$. The required sample size was then estimated using those parameters and a potential power of 0.9. A total of 86 participants was estimated to be required. We added 25% to the sample estimation to account for incomplete questionnaires, obtaining a new total of 108 participants. The survey was distributed via email lists of Finnish universities and completed by 111 participants, whose ages

ranged from 17 to 78 (68% female, 28% male). All participants were Finnish with Finnish parents. The sample size and power analysis were performed using G*Power 3.1 (Faul et al., 2007).

Statistical Analysis

To project the results onto a bidimensional SCM map, we calculated the median value of each dimension. Once candidate groups were selected, non-parametric hypothesis tests were performed to confirm that differences between groups were significant. Specifically, we used Wilcoxon signed-rank tests for the comparisons with a Dunn test for post-hoc. Additionally, all the results were FDR-corrected. The same method was used to confirm that the post-test questionnaire results from the fMRI experiment were comparable to the online survey.

Results

Respondents used the full range in both dimensions, competence (c) and warmth (w), through the survey (1-5, sd=0.8, median(c)=3.5, median(w)=3.25). The median values for each group are shown in Fig. S1A and Table S1. The group candidates selected were Brazilians, Finnish, Russians and Somalis. As Finnish represent the ingroup, they were considered from the beginning as part of the design, and by convention, the ingroup represents the high-competent/high-warm group (HC-HW); even though, some other groups were rated as more competent and warmer.

From the statistical results (see Fig. S1B-C and Table S2), we can see that all the group candidates significantly differ in one or two dimensions from each other. According to their ratings, Brazilians can be considered a medium-competent/high-warm (MC-HW) group, Russians a medium-competent/low-warm (MC-LW) group, and Somalis a low-competent/low-warm (LC-LW) group. All significant results showed a large effect size and high statistical power.

Table S 1.Competence and warmth means from the online Survey. The selected groups are marked in bold.

Nationality	Competence median (sd)	Warmth median (sd)
**Brazilian**	**3.25 (0.60)**	**3.50 (0.62)**
**Finnish**	**3.75 (0.52)**	**3.50 (0.64)**
German	4.25 (0.53)	3.50 (0.69)
Indian	3.38 (0.68)	3.50 (0.69)
Irish	3.75 (0.56)	3.75 (0.68)
Japanese	4.25 (0.48)	3.75 (0.69)
Liberian	2.75 (0.64)	3.00 (0.70)
Norwegian	4.00 (0.56)	3.75 (0.65)
Pakistani	3.00 (0.73)	3.00 (0.74)
**Russian**	**3.25 (0.62)**	**2.50 (0.75)**
**Somali**	**2.50 (0.71)**	**2.75 (0.78)**

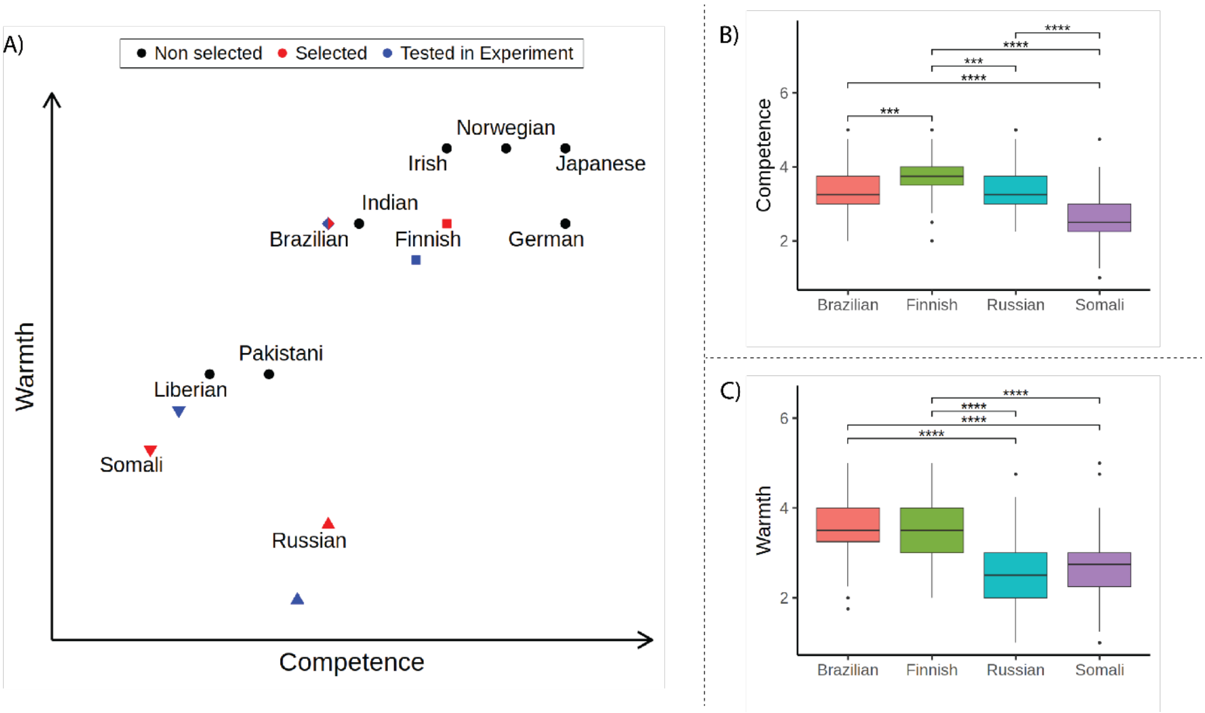

Figure S 1. Results from the online SCM survey. A) SCM map with all nationalities included. Results from the post-test questionnaire are shown in blue. The same shape is used for the same nationality. B) Results from the statistical analysis of perceived competence (****= p<0.0001, ***= p<0.001, FDR corrected). C) Results from the statistical analysis of perceived warmth (****=p<0.0001, FDR corrected).

Table S 2.Statistical results, comparison between groups

Competence

Comparison			z_value	p_value	Effect size (d)	Power (1-β)
Brazilian	<	Finnish	3.72	< 0.001	0.89	0.99
Brazilian		Russian	-0.17	0.87	-	-
Brazilian	>	Somali	-6.40	< 0.0001	1.14	0.99
Finnish	>	Russian	-3.84	< 0.001	0.87	0.99
Finnish	>	Somali	-10.07	< 0.0001	2.00	1.00
Russian	>	Somali	-6.15	< 0.0001	1.12	0.99

Warmth

Comparison			z_value	p_value	Effect size (d)	Power (1-β)
Brazilian		Finnish	-0.99	0.32	-	-
Brazilian	>	Russian	-8.86	< 0.0001	1.45	1.00
Brazilian	>	Somali	-7.86	< 0.0001	1.06	0.99
Finnish	>	Russian	-7.85	< 0.0001	1.43	1.00
Finnish	>	Somali	-6.85	< 0.0001	1.05	0.99
Russian		Somali	1.01	0.32	-	-

B. Results of the post-test questionnaire

Median values of both dimensions were calculated and projected onto a bidimensional map to form an SCM map (see Fig. S1A). All evaluations were very close to those from the online survey (difference, mean=0.125, sd=0.09). Brazilians were the only group that reported the same perceived competence and warmth in both questionnaires. The group that showed the largest difference was the Russians, who were perceived as 10% less warm than in the online survey. Additionally, the statistical results from comparisons between groups were also very similar to those from the online survey (see Fig. S2 and Tables S3-4). The main difference was that Brazilians were perceived as more competent than Russians (p=0.052) in the post-test questionnaires. The stereotypes assigned to each group were then preserved during the experiment.

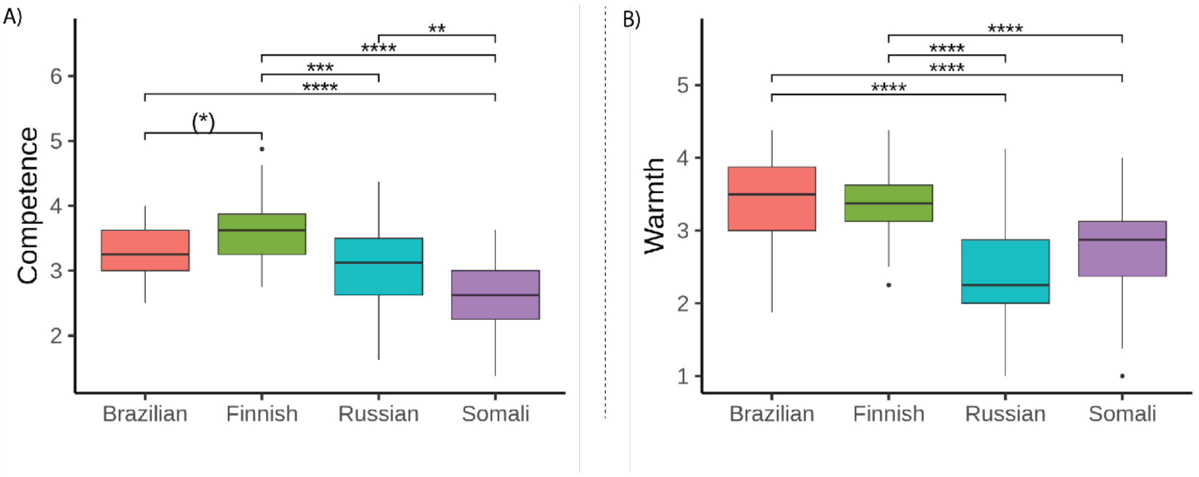

Figure S 2. Statistical results from the post-test questionnaires. Annotations: (*)=p=0.052, **=p<0.01, ***=p<0.001, ****=p<0.0001. A) Comparisons between groups of perceived competence (FDR corrected). B) Comparisons between groups of perceived warmth (FDR corrected)

Table S 3. Medians and standard deviations of post-test SCM data

Nationality	Competence median (sd)	Warmth median (sd)
Brazilian	3.25 (0.39)	3.50 (0.55)
Finnish	3.62 (0.47)	3.38 (0.48)
Russian	3.12 (0.67)	2.25 (0.81)
Somali	2.62 (0.54)	2.88 (0.58)

Table S 4. Statistical results from post-test, comparison between groups

Competence

Comparison			z_value	p_value	Effect size (d)	Power (1-β)
Brazilian	<	Finnish	2.01	0.052	0.86	0.96
Brazilian		Russian	-1.94	0.052	0.23	0.18
Brazilian	>	Somali	-5.05	< 0.0001	1.34	0.96
Finnish	>	Russian	-3.96	< 0.001	0.86	0.65
Finnish	>	Somali	-7.07	< 0.0001	1.97	0.99
Russian	>	Somali	-3.11	0.003	0.82	0.71

Warmth

Comparison			z_value	p_value	Effect size (d)	Power (1-β)
Brazilian		Finnish	-0.70	0.48	-	-
Brazilian	>	Russian	-6.37	< 0.0001	1.81	0.99
Brazilian	>	Somali	-4.70	< 0.0001	1.10	0.77
Finnish	>	Russian	-5.67	< 0.0001	1.70	0.99
Finnish	>	Somali	-4.00	< 0.0001	0.94	0.54
Russian		Somali	1.67	0.11	-	-

Note: The difference in perceived competence between Brazilians and Finnish is considered significant as the effect size and statistical power are high. Meanwhile, the difference in perceived competence between Brazilians and Russians is not significant.

C. Results interpersonal distance classifier

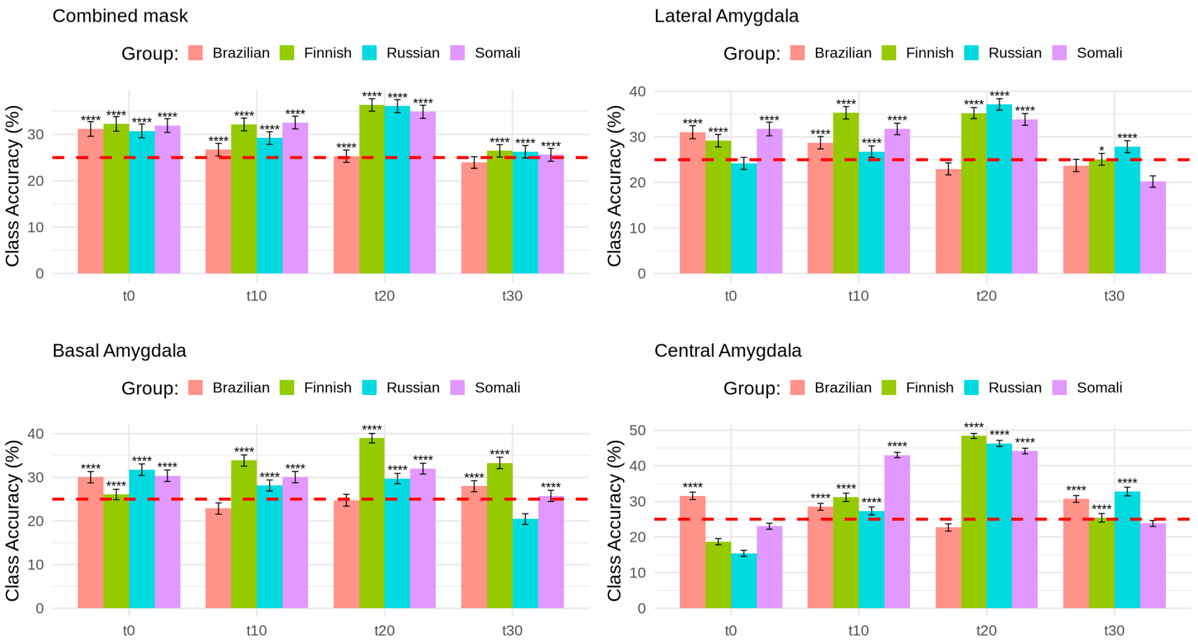

Figure S 3. Class accuracies for the interpersonal distance classifiers. Accuracies from all 16 classifiers are shown across ROIs. The red line corresponds to the chance level (0.25). Annotations: **** is used for significant accuracies above chance level (p<0.0001, Bonferroni corrected)

References

Cuddy, A. J., Fiske, S. T., Kwan, V. S., Glick, P., Demoulin, S., Leyens, J.-P., Bond, M. H., Croizet, J.-C., Ellemers, N., & Sleebos, E. (2009). Stereotype content model across cultures: Towards universal similarities and some differences. British Journal of Social Psychology, 48(1), 1–33.

Faul, F., Erdfelder, E., Lang, A.-G., & Buchner, A. (2007). G*Power 3: A flexible statistical power analysis program for the social, behavioral, and biomedical sciences. Behavior Research Methods, 39(2), 175–191. https://doi.org/10.3758/BF03193146
